# The efficacy and tolerability of bortezomib, thalidomide, and dexamethasone induction therapy with a thalidomide dose step‐up strategy in patients with newly diagnosed multiple myeloma: A prospective observational study

**DOI:** 10.1002/cnr2.2102

**Published:** 2024-05-22

**Authors:** Po‐Wei Liao, Hsueh‐Ju Lu, Tsung‐Chih Chen, Hsin‐Chen Lin, Yu‐Hsuan Shih, Chieh‐Lin Jerry Teng

**Affiliations:** ^1^ Division of Hematology/Medical Oncology, Department of Medicine Taichung Veterans General Hospital Taichung Taiwan; ^2^ Division of Hematology and Oncology, Department of Internal Medicine Chung Shan Medical University Hospital Taichung Taiwan; ^3^ School of Medicine Chung Shan Medical University Taichung Taiwan; ^4^ Department of Post‐Baccalaureate Medicine College of Medicine, National Chung Hsing University Taichung Taiwan; ^5^ Department of Life Science Tunghai University Taichung Taiwan; ^6^ Ph.D. Program in Translational Medicine National Chung Hsing University Taichung Taiwan; ^7^ Rong Hsing Research Center for Translational Medicine National Chung Hsing University Taichung Taiwan

**Keywords:** adverse events, constipation, dose intensity, overall survival, progression‐free survival

## Abstract

**Background:**

Thalidomide‐containing regimens cause adverse events (AEs) that may require a reduction in treatment intensity or even treatment discontinuation in patients with multiple myeloma. As thalidomide toxicity is dose‐dependent, identifying the most appropriate dose for each patient is essential.

**Aims:**

This study aimed to investigate the effects of a thalidomide dose step‐up strategy on treatment response and progression‐free survival (PFS).

**Methods and Results:**

This prospective observational study included 93 patients with newly diagnosed multiple myeloma (NDMM) who received bortezomib, thalidomide, and dexamethasone (VTD). The present study assessed the incidence of thalidomide dose reduction and discontinuation, the overall dose intensity, and their effects on therapeutic efficacy. Furthermore, this study used Cox proportional hazard models to analyze the factors contributing to thalidomide intolerability. The results showed the overall response rates in all patients and the evaluable patients were 78.5% and 98.7%, respectively. The median PFS in the study cohort was not reached. The most common thalidomide‐related AEs were constipation (32.3%) and skin rash (23.7%), resulting in dose reduction and discontinuation rates of 22.6% and 21.5%, respectively. The responders had a significantly higher average thalidomide dose intensity than the nonresponders (88.6% vs. 42.9%, *p* < .001).

**Conclusion:**

The thalidomide dose step‐up approach is a viable option for patients with NDMM receiving VTD induction therapy with satisfactory efficacy and tolerability. However, thalidomide intolerance may lead to dose reduction or discontinuation due to unpredictable AEs, leading to lower dose intensity and potentially inferior treatment outcomes. In addition to a dose step‐up strategy, optimal supportive care is critical for patients with multiple myeloma receiving VTD induction therapy.

## 1 INTRODUCTION

Multiple myeloma (MM) is a type of cancer that develops from abnormal plasma cells. It accounts for approximately 1.8% of all cancers and 17% of all hematological malignancies.^1^, ^2^ The incidence of MM is increasing worldwide, with the highest increase observed in Asia.^3^ MM commonly affects people aged >65 years, with a median age at diagnosis of 70 years.^4^ Over the past decade, therapeutic regimens, including proteasome inhibitors, immunomodulatory drugs, and monoclonal antibodies targeting cell surface molecules, along with autologous hematopoietic stem cell transplantation (HSCT), have significantly improved the median overall survival (OS) of patients with MM.^5^

Despite significant improvements in MM outcomes, it remains incurable. First‐line therapy offers the greatest survival benefit among the various treatments available at different stages. A more substantial treatment response leads to longer progression‐free survival (PFS) and OS.^6^ Combination therapy with bortezomib, thalidomide, and dexamethasone (VTD) is the standard of care for patients with newly diagnosed MM (NDMM) in Taiwan^7^ and several other countries.^8^ Adding an anti‐CD38 monoclonal antibody to the VTD regimen may lead to a more robust treatment response and improved PFS, particularly in transplant‐eligible patients.^9^, ^10^

However, thalidomide‐containing regimens are often associated with adverse events (AEs) such as constipation, peripheral neuropathy, and somnolence. These AEs can lead to reduced treatment intensity and treatment discontinuation. The IFM2013‐04 trial reported that up to 16% of patients discontinued thalidomide because of AEs.^11^ Given that thalidomide‐related toxicity is dose‐dependent,^12^ identifying the most appropriate dose for each individual is crucial. A dose step‐up approach is a potential solution for addressing this issue. Consequently, we conducted a prospective observational study to evaluate whether a thalidomide dose step‐up strategy could improve treatment efficacy by enhancing tolerability in patients with NDMM receiving VTD treatment.

This study aimed to examine the effects of a thalidomide dose step‐up strategy on treatment response and PFS in patients with NDMM receiving VTD induction therapy. This study also evaluated the incidence of thalidomide dose reduction and discontinuation, the overall dose intensity, and the relationship between treatment response and dose intensity. The factors associated with thalidomide intolerance were also analyzed.

## 2 METHODS

### 2.1 Patients

This single‐arm, prospective observational study aimed to analyze the clinical characteristics of patients with NDMM aged ≥20 treated between September 2016 and February 2023 at Taichung Veterans General Hospital and Chung Chan Medical University Hospital. Patients who had non‐secretory MM, tested positive for human immunodeficiency virus, had serum aspartate aminotransferase and alanine aminotransferase levels ≥ three times the upper limit of the normal range, serum bilirubin levels ≥ two times the upper limit of the normal range, Eastern Cooperative Oncology Group (ECOG) performance status >2, and ≥grade 3 peripheral neuropathy before VTD induction were excluded from this study. Finally, this analysis included 93 patients who were followed for a median duration of 1.17 years, with a follow‐up period of 0.1–5.4 years. All participants provided informed consent before participating in this study, and their details were de‐identified. This study was approved by the Institutional Review Boards of Taichung Veterans General Hospital (CF16166B) and Chung Shan Medical University Hospital (CS2‐21060) under the current version of the Declaration of Helsinki.

### 2.2 Treatments

The VTD induction regimen in our study consisted of 28‐day cycles, with weekly subcutaneous bortezomib at 1.3 mg/m^2^ and dexamethasone at 20 mg for patients aged ≥65 years and 40 mg for those aged <65 years. Patients eligible for transplantation received autologous HSCT after four cycles of VTD induction therapy, followed by four cycles of VTD consolidation therapy. Those not eligible for transplantation were administered eight cycles of VTD induction therapy. Furthermore, thalidomide maintenance therapy was administered to all patients, contingent on tolerability. In the thalidomide dose step‐up strategy, thalidomide was initiated at 50 mg/day during the first VTD induction cycle. From the second cycle onward, the thalidomide dose was increased to 100 mg/day in patients who tolerated it. For patients intolerant to thalidomide at 100 mg/day, the dose was reduced to 50 mg/day if they experienced grade 2 thalidomide‐related AEs. Thalidomide was discontinued in patients who experienced ≥ grade 3 thalidomide‐related AEs until the AEs improved to ≤ grade 2, at which point, a dose of 50 mg/day was resumed.

Participants were withdrawn if they experienced any of the following events during treatment: death, disease progression, loss of follow‐up, or noncompliance with the protocol, which was defined as a treatment delay of >4 weeks for medical or nonmedical reasons.

### 2.3 Outcome measures

This study assessed M protein and serum immunoglobulin levels bi‐monthly. The final response evaluation was performed after completing the four‐cycle VTD induction therapy. Given the study's observational nature, bone marrow examinations were not systematically conducted to assess treatment response. Instead, treatment response and disease progression were determined using the 2016 International Myeloma Working Group uniform response criteria for MM.^13^ “Evaluable patients” who adhered to the VTD regimen without any protocol deviations were selected from the entire cohort to assess their response. PFS was defined as the duration from the initiation of VTD induction therapy to disease progression or death for any reason.

AEs associated with thalidomide were evaluated following each treatment cycle using the National Cancer Institute Common Terminology Criteria for Adverse Events version 4 for grading. Thalidomide intolerance was identified when a reduction or discontinuation of thalidomide occurred due to AEs within the initial four cycles of induction therapy. Patients who were administered the full intended dose of thalidomide (50 mg) in the first cycle, followed by 100 mg in the next three cycles without any adjustments or discontinuations, were deemed to have complied with the planned dosage. The dose intensity rate was computed by dividing the thalidomide dose patients received by the prescribed amount. Thalidomide dose intensity was compared across patients with various response levels to evaluate the link between dose intensity and treatment outcomes. These outcomes were subsequently externally validated compared to the findings from the IFM‐2013‐04 trial.^11^ Further analysis was performed to identify potential factors influencing the tolerability of VTD induction therapy in patients with NDMM.

### 2.4 Statistical analyses

Categorical variables were analyzed using the chi‐square or the Fisher exact test, whereas continuous variables were analyzed using the Mann–Whitney *U* test. The Kaplan–Meier method was used to calculate PFS, and differences were compared using the log‐rank test. Cox proportional hazard models were used to identify factors associated with thalidomide dose and PFS, with results reported as hazard ratios accompanied by 95% confidence intervals. Statistical significance was defined as *p* < .05. All statistical analyses were performed using the SPSS software package (version 22.0; SPSS, Inc., Chicago, IL, USA).

## 3 RESULTS

### 3.1 Patients and treatments

**FIGURE 1:**
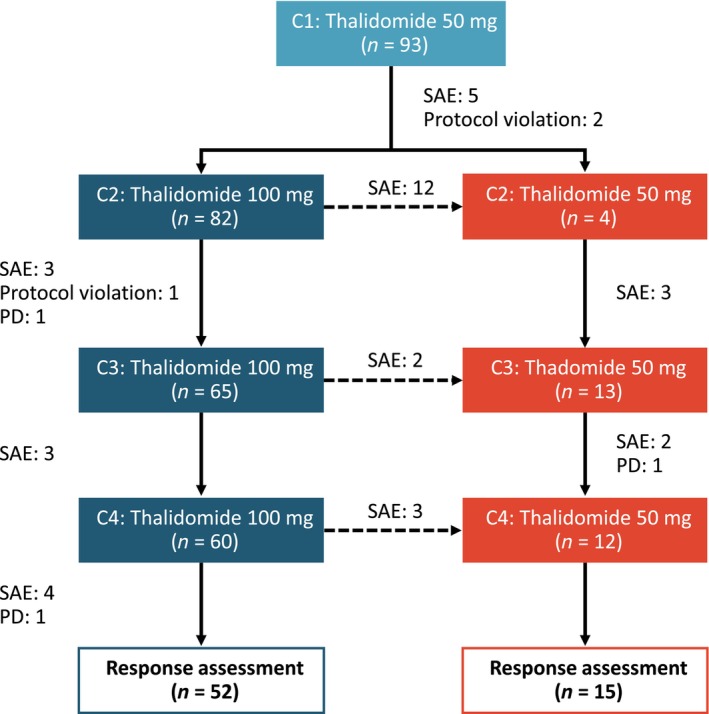
The study flow. PD, progressive disease; SAE, severe adverse event.

Table 1 shows the clinical characteristics of the study cohort. The median age of the patients was 65 years, and the most common subtype of MM was IgG, accounting for 49.5% of patients. Furthermore, 28.0% of the patients had renal function impairment at diagnosis, characterized by a serum creatinine level >2 mg/dL. Throughout the treatment course, 21 patients could not tolerate the AEs of thalidomide at a dosage of 100 mg/day, and 20 patients discontinued thalidomide owing to severe AEs. Additionally, three patients withdrew from the study due to disease progression, and another three left due to protocol violations (Figure 1).

**TABLE 1:** Patients' characteristics (*n* = 93).

Age, median (range, years)	65	(33–93)
Sex, no. (%)		
Male	47	(50.5)
Female	46	(49.5)
ECOG performance status, no. (%)		
0–1	86	(92.5)
≥2	7	(7.5)
Type of myeloma, no. (%)		
IgG	46	(49.5)
IgA	21	(22.6)
IgM	1	(1.1)
Light chain	25	(26.9)
R‐ISS disease stage,^a^ no. (%)		
I	14	(16.7)
II	50	(59.5)
III	20	(23.8)
Cytogenetic abnormalities,^a^ no. (%)		
t(4;14)	10/84	(11.9)
17p deletion	24/84	(28.6)
t(14;16)	2/84	(2.4)
β2‐microglobulin level, no. (%)		
<3.5 mg/L	34	(36.5)
3.5–5.5 mg/L	26	(28.0)
>5.5 mg/L	33	(35.5)
Renal impairment,^b^ no. (%)		
Yes	26	(28.0)
No	67	(72.0)

Abbreviations: ECOG, Eastern Cooperative Oncology Group; R‐ISS, Revised International Staging System.

^a^ 84 patients were evaluable; cytogenetics was obtained by fluorescence in situ hybridization. Patients could have more than one abnormality.

^b^ Renal impairment was characterized by a serum creatinine level >2 mg/dL.

### 3.2 Efficacy assessment

**FIGURE 2:**
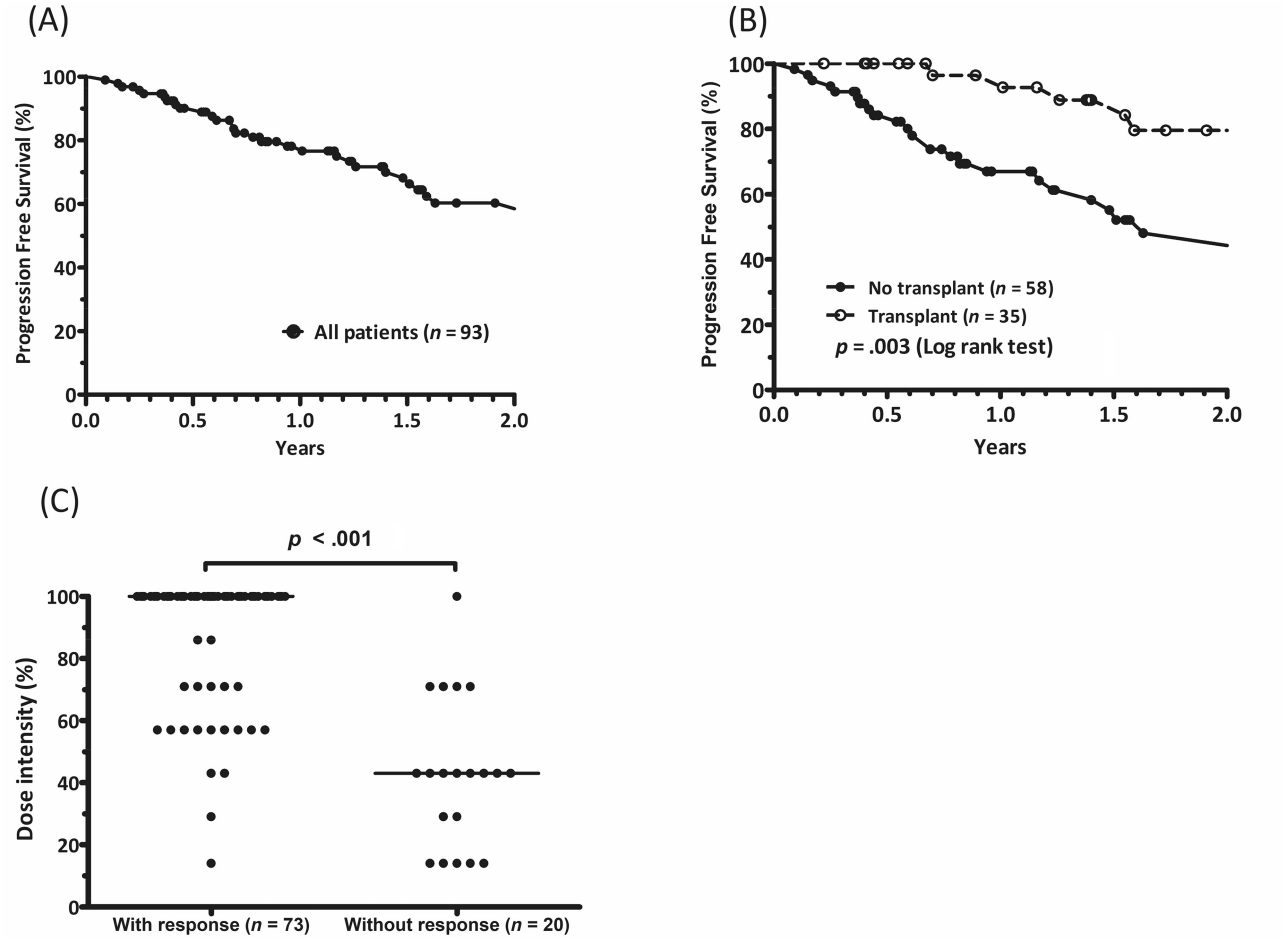
Assessment of efficacy (A) median progression‐free survival (PFS) of the study cohort was not reached. (B) Patients who received bortezomib, thalidomide, and dexamethasone induction followed by autologous hematopoietic stem cell transplantation (HSCT) had a longer median PFS than those who did not undergo autologous HSCT (not reached vs. 19.5 months, *p* = .003). (C) Responders had a significantly higher average thalidomide dose intensity than nonresponders (88.6% vs. 42.9%, *p* < .001). *Lines indicate median values for each group.

This study found that the overall response rates in all patients and the evaluable patients were 78.5% and 95.7%, respectively. For all patients, ≥ partial response (PR) and very good PR (VGPR) rates were 78.5% and 51.6%, respectively. For evaluable patients, ≥ PR and ≥VGPR rates were 95.7% and 62.9%, respectively (Table 2). In terms of PFS, the median PFS for the study cohort was not achieved (Figure 2A). Patients who underwent VTD induction followed by autologous HSCT had a longer median PFS than those who did not undergo autologous HSCT (not reached vs. 19.5 months, *p* = .003) (Figure 2B).

**TABLE 2:** Treatment response.

	All patients (*n* = 93)	Evaluable patients (*n* = 70)
After two cycles, *n* (%)		
≥VGPR	40 (43.0)	36 (51.4)
≥PR	78 (83.9)	68 (97.1)
After four cycles, *n* (%)		
≥VGPR	48 (51.6)	44 (62.9)
≥PR	73 (78.5)	67 (95.7)

Abbreviations: PR, partial response; VGPR, very good partial response.

### 3.3 Thalidomide‐associated adverse events and dose reduction

Table 3 shows AEs associated with thalidomide use. The leading thalidomide‐related AEs were constipation (32.3%, 30/93) and skin rash (23.7%, 22/93), which were also the most common grade ≥3 AEs (11.8%, constipation; 10.8%, skin rash). In contrast, peripheral neuropathy was observed in only 9.7% (9/93) of patients. However, acyclovir prophylaxis was not mandated in this study, which potentially explains the varied incidence of herpes zoster noted in our study cohort.

**TABLE 3:** Adverse events during the VTD induction.

Adverse events, *n* * (%)	All patients (*n* = 93)				Transplant (+) (*n* = 35)				Transplant (−) (*n* = 58)			
	Any grade		≥Grade 3		Any grade		≥Grade 3		Any grade		≥Grade 3	
Hematological												
Neutropenia	12	(12.9)	3	(3.2)	5	(14.3)	2	(5.7)	7	(12.1)	1	(1.7)
Anemia	8	(8.6)	1	(1.1)	1	(2.9)	0		7	(12.1)	1	(1.7)
Thrombocytopenia	8	(8.6)	0		3	(8.6)	0		5	(8.6)	0	
Nonhematological												
Constipation	30	(32.3)	11	(11.8)	11	(31.4)	3	(8.6)	19	(32.8)	8	(13.8)
Skin rash	22	(23.7)	10	(10.8)	10	(28.6)	3	(8.6)	12	(20.7)	7	(12.1)
Peripheral edema	13	(14.0)	5	(5.4)	3	(8.6)	0		10	(17.2)	5	(8.6)
Herpes zoster	9	(9.7)	5	(5.4)	1	(2.9)	0		8	(13.8)	5	(8.6)
Peripheral neuropathy	9	(9.7)	3	(3.2)	2	(5.7)	1	(2.9)	7	(12.1)	2	(3.4)
Infection	8	(8.6)	1	(1.1)	1	(2.9)	1	(2.9)	7	(12.1)	0	
Gastrointestinal symptoms	5	(5.4)	1	(1.1)	2	(5.7)	0		3	(5.2)	1	(1.7)
Somnolence	3	(3.2)	2	(2.2)	1	(2.9)	1	(2.9)	2	(3.4)	1	(1.7)
Thrombosis	1	(1.1)	1	(1.1)	0		0		1	(1.7)	1	(1.7)

Abbreviation: VTD, bortezomib, thalidomide, and dexamethasone.

* Patients could have more than one adverse event.

The thalidomide dose was adjusted according to the observed AEs. In the entire study cohort, 54 of 93 (58.1%) participants received a planned dose of thalidomide. Dose reduction and discontinuation rates were 22.6% and 21.5%, respectively. The overall dose intensity in the study population was 80.0% (Table 4).

**TABLE 4:** Thalidomide dose modification.

	Our study			IFM2013‐04
	All (*n* = 93) (%)	Transplant (−) (*n* = 58) (%)	Transplant (+) (*n* = 35) (%)	Transplant‐eligible (*n* = 169) (%)
Dose intensity^a^	80.0	73.7	90.9	89.9
Dose reduction	22.6	27.6	14.3	21.3
Discontinuation	21.5	36.2	14.3	16.0
100% of planned dose	58.1	50.0	71.4	62.7

^a^ Patients with protocol violations were not included.

### 3.4 Patients with lower thalidomide intensity had worse treatment responses

We compared the thalidomide dose intensities among all patients with different response depths. The results indicated that responders had a significantly higher average thalidomide dose intensity than non‐responders (88.6% vs. 42.9%, *p* < .001) (Figure 2C). In addition, patients with ≥ VGPR (88.4% vs. 42.9%, *p* < .001) and ≥ PR (89.1% vs. 42.9%, *p* < 0.001) had a higher average thalidomide dose intensity than those who did not respond to treatment.

### 3.5 Factors associated with patients' tolerability

We further investigated the potential factors associated with the tolerability of VTD induction in patients with NDMM. However, we found no significant associations between thalidomide dose reduction or discontinuation and age, sex, ECOG performance status at diagnosis, MM type, beta‐2‐microglobulin levels, high‐risk cytogenetics, or renal function impairment (Table 5).

**TABLE 5:** Factors affecting the patients' tolerability to thalidomide.

	Simple model		
	HR	(95% CI)	*p* Value
Age			
≤65 years	Reference		
>65 years	1.9	(0.8–4.3)	.154
Sex			
Female	Reference		
Male	2.0	(0.9–4.7)	.107
ECOG performance status			
0–1	Reference		
≥2	4.4	(0.8–24.2)	.086
Type of myeloma			
IgG	Reference		
IgA	1.1	(0.4–3.1)	.929
IgM	0		1
Light chain	1.3	(0.5–3.6)	.562
R‐ISS stage			
I	Reference		
II	1.5	(0.4–5.6)	.518
III	1.7	(0.4–7.2)	.494
Serum β2‐microglobulin level			
<3500 ng/mL	Reference		
3500–5500 ng/mL	1.3	(0.5–3.8)	.580
>5500 ng/mL	1.2	(0.4–7.2)	.729
High‐risk cytogenetics			
0	Reference		
≥1	0.7	(0.3–1.7)	.401
Renal impairment			
No	Reference		
Yes	1.9	(0.8–4.8)	.167

Abbreviations: CI, confidence interval; ECOG, Eastern Cooperative Oncology Group; ISS, International Staging System; HR: hazard ratio.

## 4 DISCUSSION

In this prospective observational study, we investigated the efficacy and tolerability of a thalidomide dose step‐up strategy in patients with NDMM receiving VTD induction therapy. Our findings showed that this approach achieved a favorable overall response rate and PFS without significant incidence of treatment discontinuation, indicating appropriate tolerability. However, we also found that the intolerability of this approach was unpredictable, underscoring the importance of regular monitoring of patients with NDMM receiving the VTD regimen.

A dose step‐up approach has been commonly used in early phase clinical studies to determine the optimal dose, schedule, or both.^14^ Proper dose escalation with frequent monitoring of AEs can significantly enhance patient safety during anticancer treatment.^15^ The use of thalidomide in MM treatment has been associated with several AEs, including teratogenicity, peripheral neuropathy, somnolence, constipation, dermatological problems, and thromboembolic complications.^16^ Remarkably, over 20% of the patients who received VTD induction therapy with a daily dose of 100 mg thalidomide experienced ≥ grade 3 peripheral neuropathy,^17^ which easily led to treatment discontinuation. Our study's dose reduction and discontinuation rates were 22.6% and 21.5%, respectively. This was a single‐arm, prospective, observational study, so internal group comparison was impossible. To evaluate patient tolerability using this approach, we compared dose intensity, reduction, and discontinuation between our transplant cohort and patients from the IFM‐2013‐04 trial (Table 4).^11^ The comparable data suggest that our real‐world thalidomide dose step‐up strategy was well‐tolerated compared with clinical trial data.

Interestingly, in our study, constipation but not peripheral neuropathy was the leading cause of thalidomide dose reduction and discontinuation. This result is inconsistent with the findings from other clinical trials,^11^, ^18^ and the underlying mechanisms for this discrepancy remain unclear. However, a higher body mass index is a risk factor for chemotherapy‐induced peripheral neuropathy,^19^ and Asian patients usually have a lower body mass index than Western patients. Underreporting may explain these differences. In addition, we observed different incidences of various AEs between Western and Asian patients receiving thalidomide treatment owing to different genetic backgrounds. For example, Asian patients exhibit fewer thromboses.^20^ Further studies are required to confirm this hypothesis.

Responders in our study showed significantly higher thalidomide intensity than nonresponders, implying that thalidomide intolerance may lead to inferior outcomes in patients with NDMM receiving VTD induction therapy. This phenomenon has been observed in other hematological malignancies, as demonstrated in a study by Bosly et al.,^21^ who reported that patients with diffuse large B‐cell lymphoma who received ≥90% of the average relative dose intensity of the CHOP‐21 regimen had a significantly better OS than those who received <90% of the average relative dose intensity. This finding underscores the importance of improving thalidomide tolerability in MM, as it may significantly affect patient outcomes. Unfortunately, thalidomide intolerance remains unpredictable, and proactive measures are necessary to mitigate its effects. Supportive care and frequent monitoring can reduce the incidence of AEs, facilitate increased dose intensity, and improve patient outcomes.

The single‐arm design was the primary limitation of this study. We compared our data with those of other trials to overcome this limitation. In the IFM2013‐04 trial, 66.3% of the patients in the VTD arm achieved at least one VGPR, whereas the ≥ VGPR rate in our study was 62.9%. To fully evaluate the efficacy and safety of the thalidomide dose step‐up and fixed‐dose approaches, a randomized controlled study is necessary. Moreover, owing to the lack of routine bone marrow examinations for response assessment, stringent complete response could not serve as an outcome measure. Another limitation is the potential contribution of bortezomib‐associated AEs to the interruption or intolerance of thalidomide therapy, which may not have been assessed in the current study. Additionally, a larger sample size is required to identify potential factors associated with thalidomide intolerance.

## 5 CONCLUSIONS

Our study demonstrated that the thalidomide dose step‐up approach is a viable option for patients with NDMM receiving VTD induction therapy, with satisfactory efficacy and tolerability. However, thalidomide intolerance may lead to dose reduction or discontinuation due to unpredictable AEs, leading to lower dose intensity and potentially inferior treatment outcomes. Our study also revealed that constipation was the leading AE, compared to other studies in which peripheral neuropathy was more prevalent in patients receiving the VTD regimen. In addition to the dose step‐up strategy, optimal supportive care is critical for patients with MM receiving VTD induction therapy.

## AUTHOR CONTRIBUTIONS

**Po‐Wei Liao:** Conceptualization; writing – original draft; investigation. **Hsueh‐Ju Lu:** Conceptualization; investigation; validation. **Tsung‐Chih Chen:** Formal analysis; investigation. **Hsin‐Chen Lin:** Methodology; data curation. **Yu‐Hsuan Shih:** Methodology; data curation. **Chieh‐Lin Jerry Teng:** Conceptualization; methodology; funding acquisition; writing – original draft; writing – review and editing.

## CONFLICT OF INTEREST STATEMENT

Chieh‐Lin Jerry Teng received honorarium and consulting fees from Novartis, Roche, Pfizer, Takeda, Johnson and Johnson, Amgen, BMS Celgene, Kirin, and MSD. The other authors have no conflicts of interest.

### ETHICS STATEMENT

Participant details were de‐identified, and the study was approved by the Institutional Review Boards of Taichung Veterans General Hospital (CF16166B) and Chung Shan Medical University Hospital (CS2‐21060) in accordance with the current version of the Declaration of Helsinki.

### PATIENT CONSENT STATEMENT

All participants provided informed consent before participating in this study, and their details were de‐identified.

## Data Availability

The data that support the findings of this study are available from the corresponding author upon reasonable request.
